# Head and eyes: Looking behavior in 12- to 24-month-old infants

**DOI:** 10.1167/jov.21.8.18

**Published:** 2021-08-17

**Authors:** Jeremy I. Borjon, Drew H. Abney, Chen Yu, Linda B. Smith

**Affiliations:** 1 Department of Psychological and Brain Sciences, Indiana University, Bloomington, IN, USA; 2 Department of Psychology, University of Georgia, Athens, GA, USA; 3 Department of Psychology, University of Texas, Austin, TX, USA; 4 School of Psychology, University of East Anglia, East Anglia, UK

**Keywords:** infant vision, active vision, attention, head-eye alignment, motor development, sensorimotor coordination

## Abstract

This study demonstrates evidence for a foundational process underlying active vision in older infants during object play. Using head-mounted eye-tracking and motion capture, looks to an object are shown to be tightly linked to and synchronous with a stilled head, regardless of the duration of gaze, for infants 12 to 24 months of age. Despite being a developmental period of rapid and marked changes in motor abilities, the dynamic coordination of head stabilization and sustained gaze to a visual target is developmentally invariant during the examined age range. The findings indicate that looking with an aligned head and eyes is a fundamental property of human vision and highlights the importance of studying looking behavior in freely moving perceivers in everyday contexts, opening new questions about the role of body movement in both typical and atypical development of visual attention.

## Introduction

Gaze is directed to select targets and is maintained on selected targets to gather relevant information. Thus looking behavior across the lifespan is intensely studied (e.g., Aslin, 2007; Ballard & Hayhoe, 2009; Brams, Ziv, Levin, Spitz, Wagemans, Mark Williams, & Helsen, 2019; Oakes, 2015). However, there is still a great deal not known about looking behavior in freely moving individuals in the purposeful tasks of everyday life (Jovancevic-Misic & Hayhoe, 2009; Lappi, 2016; Schmitow, Stenberg, Billard, & Hofsten, 2013; Tatler, Hayhoe, Land, & Ballard, 2011). This lack of knowledge poses a significant barrier to research on a current topic of interest in developmental science: the ability of newly autonomous toddlers to maintain gaze on a single object in the context of natural play is increasingly implicated as both a biomarker and training ground for later development of the executive functions mediated by the prefrontal cortex (Brandes-Aitken, Braren, Swingler, Voegtline, & Blair, 2019; Fisher, 2019; Rosen, Amso, & McLaughlin, 2019; Werchan & Amso, 2017; Yu & Smith, 2016). Because active looking involves both head and eyes, we used head-mounted eye-tracking and motion-capture sensors to quantify eye and head movements in 12- to 24-month-old toddlers as they actively interacted with and directed gaze to objects during play. The main finding is that the duration of gaze to an object, be it brief or sustained, was *synchronous with* decreased head movement. The findings open new questions about the role of body movement in both typical and atypical development of visual attention.

Directing gaze to a target selectively supports visual processing of that target over other information because the retinal area around the gaze point captures a higher resolution image than does the periphery (Dowling, 1987; Lee, 1996; May, 2006; Meister & Tessier-Lavigne, 2013). Thus, when a perceiver sustains gaze on a target, they optimize the extraction of visual information from the target relative to the periphery. The eyes, however, do not operate in isolation. Eyes are located in a head, which is on a body, all of which can move independently. Therefore stabilizing gaze on a target depends on coordinating eye and head movements (Crawford, Henriques, & Medendorp, 2011; Kretch & Adolph, 2015; Nakagawa & Sukigara, 2013; Regal, Ashmead, & Salapatek, 1983). Active purposeful vision, from making a sandwich (Hayhoe, Shrivastava, Mruczek, & Pelz, 2003) to putting one toy on top of another (Yu & Smith, 2012), often includes large head movements that can be both goal-directed and compensatory to actions such as reaching or posture change (Bertenthal & Von Hofsten, 1998; von Hofsten & Rosander, 2018; Von Hofsten, Vishton, Spelke, Feng, & Rosander, 1998). The central goal of the present study was to quantify head and eye coordination and sustained gaze in freely moving infants 12 to 24 months of age. This is the developmental period during which active object play strongly predicts long-term outcomes in executive function and self-regulation (Rosen et al., 2019; Werchan & Amso, 2017).

It is well known that freely moving perceivers, both adults and infants, are strongly biased to direct their gaze toward targets with their eyes and head aligned, turning both the eyes and head in the same direction to the target (Bambach, Crandall, Smith, & Yu, 2018; Bambach, Smith, Crandall, & Yu, 2016; Foulsham, Walker, & Kingstone, 2011; Kretch & Adolph, 2015; Solman, Foulsham, & Kingstone, 2017; Tatler et al., 2011; van Renswoude, van den Berg, Raijmakers, & Visser, 2019; Yoshida & Smith, 2008; Yu & Smith, 2012). When perceivers shift their gaze to a new target, the eyes and head are misaligned for typically less than 500 milliseconds, as either the eyes shift first, followed by the head (typical in adults, Corneil, 2012; Doshi & Trivedi, 2012; Nakashima & Shioiri, 2014), or the head shifts first followed by eyes (frequent in infants and children, Bloch & Carchon, 1992; Funk & Anderson, 1977; Nakagawa & Sukigara, 2013; Regal et al., 1983; Schmitow, Stenberg, Billard, & Hofsten, 2013; Tronick & Clanton, 1971). Notably for natural movements in adults, if planning is possible, the head will frequently move ahead of the eyes (Hayhoe, 2009). Once the shift is accomplished, the extant evidence suggests that purposeful looks occur with eyes and head pointed roughly in the same direction, the perhaps energetic “resting state” for gaze (Seemiller, Port, & Candy, 2018; van Renswoude et al., 2019). This bias is also evident in the spatial distribution of gaze captured by head-mounted eye-trackers which find gaze to be pervasively centered in the head-centered field of view (Bambach et al., 2016; Y. Li, Fathi, & Rehg, 2013).

The present study focuses on eye-head coordination and gaze duration in 12- to 24-month old infants because sustained gaze on an object during this period strongly predicts cognitive development more generally; from individual differences in visual attention (Lansink & Richards, 1997; Richards & Casey, 1992; Ruff, 1986), to differences in self-regulation and self-control (Kochanska, Murray, & Harlan, 2000; Reck & Hund, 2011; Ruff, 1986), as well as language development (Welsh, Nix, Blair, Bierman, & Nelson, 2010; Yu, Suanda, & Smith, 2019) and later school achievement (Kannass, Oakes, & Shaddy, 2006; Ruff & Lawson, 1990). Visual attention in active and unrestrained toddlers has been characterized as recruiting the whole body. Toddlers often both move their body closer to objects and hold objects close to their body while looking (Richards & Cameron, 1989; Richards & Casey, 1992; Ruff & Lawson, 1990; Ruff & Rothbart, 1996; Yu & Smith, 2012). During this period of rapid physical growth, infants are also just beginning to control their bodies and have well-documented difficulties in stabilizing their head (Bertenthal & Von Hofsten, 1998; Flatters, Mushtaq, Hill, Rossiter, Jarrett-Peet, Culmer, Holt, Wilkie, Mon-Williams, 2014; Ledebt & Bril, 2000) especially during large body movements, the common context for toddler everyday vision (Adolph, Vereijken, & Shrout, 2003; Bertenthal & Von Hofsten, 1998; Claxton, Haddad, Ponto, Ryu, & Newcomer, 2013; Claxton, Melzer, Ryu, & Haddad, 2012; Claxton, Strasser, Leung, Ryu, & O'Brien, 2014; Flatters et al., 2014; von Hofsten & Rosander, 2018; Von Hofsten et al., 1998). Toddlers, like adult perceivers, primarily direct gaze to the center of the head-center field of view (Bambach et al., 2016). However, the field lacks precise quantification of the relations among eye-head coordination, head stabilization, and gaze to an object in freely moving toddlers during active engagement with objects.

The starting hypothesis is that head *stabilization* is strongly associated with maintained gaze to an object. This hypothesis is suggested by classic studies on focused attention in late infancy (Ruff & Capozzoli, 2003; Ruff, Capozzoli, & Weissberg, 1998; Ruff & Rothbart, 1996). These studies found that long looks by toddlers during object play were associated with a stilled head. In these earlier studies, look durations and head movements were measured by human coders. Here, wearable sensors are used to provide more precise temporal spatial measures of the hypothesized decrease in head movements during gaze to an object. The interest in head movements and gaze duration is also motivated by research on atypically developing children that has shown an association between large head movements during a purposeful task and poor attentional control (Klingberg, Forssberg, & Westerberg, 2002; F. Li et al., 2016; Teicher, Ito, Glod, & Barber, 1996). Together, these observations suggest that *maintaining* gaze to a target is accompanied by an aligned head and eyes and decreased head movements *during the look* to an object.

## Methods

### Participants

A total of 44 infants (22 male) participated in multiple testing sessions when they were 12, 15, 18, 21, or 24 months of age. Infants possessed no reported visual-acuity or binocular-vision abnormalities. This period of development is under study because of the focus of recent work on sustained attention and its role as a predictor of later developmental outcomes (Brandes-Aitken et al., 2019; Reck & Hund, 2011; Yu et al., 2019). There are no specific a priori developmental hypotheses, but the broad age range spans a period of marked changes in general sensory-motor skills (Adolph & Franchak, 2017; Libertus & Hauf, 2017; McGraw, 2004; Soska, Robinson, & Adolph, 2015) and is also characterized by the overall shortening of look durations to objects (Bronson, 1991; Colombo, Mitchell, Coldren, & Freeseman, 1991; Helo, Rämä, Pannasch, & Meary, 2016; Wass & Smith, 2014). Both factors could be relevant to the role of head and eye coordination in sustained gaze to an object. Each infant participated at different ages for an average of 2.49 sessions (*SD* = 1.16) yielding a total of 107 sessions distributed across the five ages at testing. Table 1 shows the data for the sessions contributed by each participant. The sample of infants was broadly representative of Monroe County, Indiana (84% European American, 5%African American, 5% Asian American, 2% Latino, 4% other) and consisted of predominantly working- and middle-class families. All research was approved by the Human Subjects and Institutional Review Board at Indiana University (Protocol no. 0808000094) and adhered to the tenets of the Declaration of Helsinki. Caregivers volunteering their infants for the study were fully informed of the study procedures and completed written informed consent and permission forms in advance of the study.

**Table 1.** Breakdown of subject participation for each age level. Age at which subject was tested, with ‘x’ indicating when tested.

Subject no.	12 Month	15 Month	18 Month	21 Month	24 Month
1					x
2		x			
3		x	x		
4			x	x	x
5				x	
6	x	x	x	x	x
7	x	x		x	x
8	x	x			x
9	x	x		x	x
10	x		x		x
11			x	x	x
12				x	x
13		x		x	x
14		x		x	
15	x	x		x	
16				x	x
17			x	x	x
18	x	x			
19	x	x	x	x	x
20				x	
21			x	x	x
22					
23			x	x	x
24	x	x	x	x	x
25	x		x	x	x
26	x	x			
27					x
28	x		x	x	x
29		x	x	x	x
30	x	x			
31	x				
32	x	x			
33	x				
34	x	x			
35	x	x			
36	x				
37	x		x	x	x
38		x			
39			x	x	x
40				x	x
41			x		x
42			x		
43			x		x
44			x		x
Total	20	19	19	23	26

### Stimuli

There were 30 novel objects constructed in the laboratory and pilot-tested to be interesting and engaging to infants. Each object consisted of multiple parts (some moveable) and were of similar size (∼280 cm^3^) and weight (∼95 g). A unique subset of six objects were chosen for use in each session and were organized into two sets of three. Each object in the set of three had a unique uniform color (red, blue, green). At each age level, repeating participants received a different set of toys so that no child experienced a repeated set of toys during their participation in the study.

### Experimental setup

**Figure 1.**
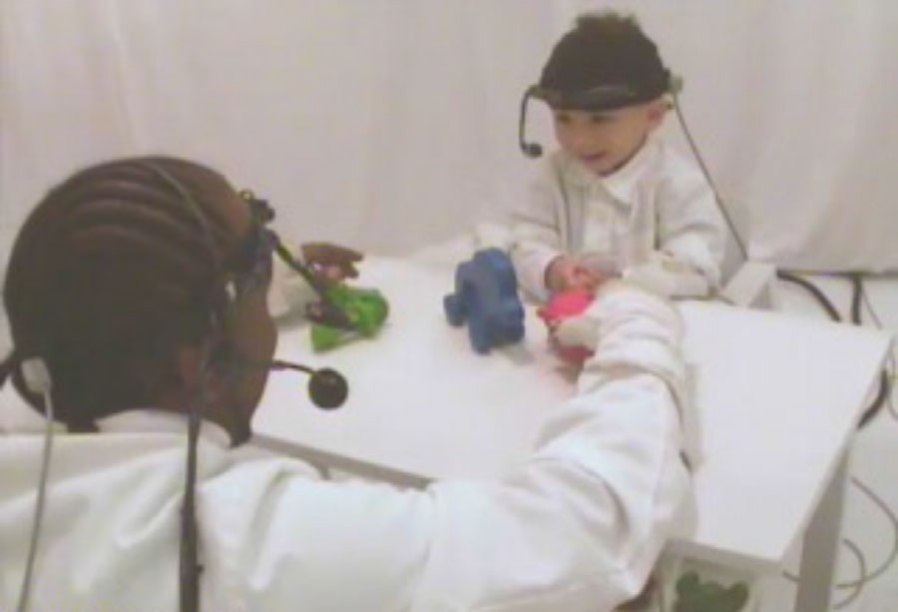
Experimental setup.

Infants sat at a small table (61 cm × 91 cm × 64 cm) while their caregiver sat across the table from them (Figure 1). The infant was free to shift, lean, rotate the upper body and head, and reach for objects in play on the tabletop. The infant wore a head-mounted eye-tracker (Positive Science, LLC, Rochester, NY, USA) designed for use with infants. The tracking system included two cameras: (1) an infrared camera mounted on the head and pointed to the right eye of the participant to record eye images and (2) a scene camera that captures the events from the participant's perspective. The scene camera's visual field has a diagonal of 108°, providing a broad view to approximate the full visual field. The eye-tracking system recorded both the egocentric view video and eye-in-head position (x and y coordinates) in the captured scene at a sampling rate of 30 Hz. A wired motion capture sensor was affixed to the eye-tracker on the right temple of the infant's head (Polhemus Liberty; Polhemus, Colchester, VT, USA). The motion-capture sensor collected rotational position data (roll, pitch, and yaw) at 60 Hz.

Placing the head gear and eye-tracker calibration before entering the testing room, in the waiting area, a first experimenter desensitized the infant to touches to the head and hair by lightly touching the hair several times when the interest of the infant was directed to a toy. Both the caregiver and the infant entered the experimental room, and a second experimenter and the caregiver engaged the infant with toys not used in the experiment. The infant's head gear was placed while the infant was engaged with the toy. The first experimenter then adjusted the scene camera to ensure the scene camera captured the caregiver across the table and also the manual actions of the infant. The overall success rate for infant wearing of the sensors and calibration is more than 70% (see Slone, Abney, Borjon, Chen, Franchak, Pearcy, Suarez-Rivera, Xu, Zhang, Smith, Yu, 2018).

### Instructions and procedure

Caregivers were told the goal of the experiment was to study how infants manually and visually explored novel objects and that they should encourage their infants to interact with the objects as naturally as possible. Each of the two sets of objects were played with twice for 1.5 minutes, resulting in six minutes of play data per session.

### Data processing

During post-processing and before coding, the quality of the eye-tracking video for each infant was checked to ensure the quality of calibration at the end, as well as the beginning, of the session. If necessary, manual recalibration was conducted by identifying moments in which the pupil and corneal reflection are accurately detected, and the eye is stably fixated on a clearly identifiable point in space in the scene image. These locations were chosen as re-calibration points. For a more in-depth discussion of the calibration and recording procedure (see Slone et al, 2018).

#### Looking

Within the study of vision, operational definitions of oculomotor functions such as saccades and fixations vary (Hessels, Niehorster, Nyström, Andersson, & Hooge, 2018) and there have been many debates about space-based versus object-based characterizations of attention (Chen, 2012; Logan, 1996; Scholl, 2001). The present study used object-based measures of attention because it is a better indicator of the duration of visual attention to a target when the targets are three-dimensional objects in a three-dimensional space and the perceiver is moving. In addition, gaze to an object (not a spatial location) has been the principal measure of sustained attention in studies of freely moving toddlers. Accordingly, *looks* to objects were measured in terms of continuous gaze that fell on an object.

The three regions-of-interest (ROIs) were defined in the head-camera videos as each of the three different and uniformly colored objects. ROI coding was done by highly trained coders who were responsible for coding many different projects and were naïve to the specific hypotheses or experimental questions of this study. Each of the three ROIs was coded separately. Frame-by-frame coders marked when the crosshair indicating gaze fell on a pixel of the object. This was a relatively easy task as each object was a unique color and the experimental room was white and both parent and child wore white smocks. Eye images were rendered via picture-in-picture superimposition at the upper-right corner of a scene frame, which allowed coders to constantly use the eye images as a reference to verify reliability of the crosshair indicating gaze direction in view. If coders detected that the eye-tracking software failed to detect the pupil correctly due to image quality or eye blinks, coders disregarded that frame. An unbroken look was defined as one that fell within a single object (Slone et al., 2018) and lasted a minimum of 15 frames, corresponding to 500 ms (Yu & Smith, 2012). This definition of a look thus includes both saccades and fixations. A second coder independently coded a randomly selected 10% of the frames (111,539 frames) with the inter-coder reliability ranging from 82% to 95% (Cohen's *κ* = 0.81).

Analyses were conducted only on looks directed to one of the three objects in play. The head movements from the 44.45% of the play periods excluded from analyses were used for the determination of baseline rotational velocity of the head for each subject (described below).

#### Gaze clustering

Gaze refers to the eye-tracking data and need not be part of a look directed to an individual object. To measure the dispersion of frame-by-frame gaze across the head camera, the x-y coordinates from head-mounted eye-trackers were normalized for each individual by alignment to their centroid calculated from individual gaze points. Such an approach corrects for any off-center offset due to an imperfect positioning of the scene camera while preserving the original spread of the distribution (Bambach et al., 2018; Bambach et al., 2016; Slone et al., 2018). The Euclidean distance from each x-y coordinate of eye position to the center of the scene-camera image, the origin, was then calculated in visual degrees.

To calculate the proportion of gaze points that fell within a radius of 10° and 20° from the center, the degrees per pixel in the head camera image was first determined. Frames from the head camera video were 480 pixels in height by 640 pixels with a diagonal of 108° in visual angle (Smith, Yu, Yoshida, & Fausey, 2015). Therefore the head camera image is 86.4° in width and 64.8° in height. This results in 7.404 pixels per visual degree. For all analyses, the x-y coordinates of the head-mounted eye-tracker were converted into visual degrees by dividing the normalized x-y coordinates by 7.404.

For some analyses, looks (continuous gaze to an object) were categorized into two classes by duration: short (shorter than three seconds) or long (equal to or longer than three seconds in duration) as explained in the results section. Multivariate kernel density estimates of the normalized gaze distributions for these categorized long and short looks were independently calculated for each age and each look type using *kde2d* in Matlab and normalizing the resulting density by dividing all values by the maximum density value for that age level and look type. This resulted in a series of numbers between 0 and 1, separately calculated for each age level and look type.

### Rotational velocity

Head stabilization in infants is typically measured in terms of the rotational coordinates of the head (Ledebt & Bril, 2000; Ledebt & Wiener-Vacher, 1996; Reisman & Anderson, 1989; Richards & Hunter, 1997; Rosander & Von Hofsten, 2000; Wiener-Vacher, 1996). Participants were equipped with a wired, magnetic motion capture marker (Polhemus Liberty; Polhemus) placed on the right temple of the head to record head rotation (roll, pitch, and yaw) and position (x, y, and z) during the task, at a rate of 60 Hz. The placement of the motion sensor was not consistent between subjects during the experiment because of toddler behavior. Experimenters needed to place the sensor and adjust it in one or two moves or else the toddler would pull it off. Therefore small variation was allowed in final placement. Although the sensor is at the same location (right temple) the orientation of the sensor varies. Thus translation is an unreliable measure, and rotation was used. Rotational data were converted from millimeters to degrees by calculating the angular rotation between subsequent samples using the following formula in Matlab, where *rpy* represents an n-by-3 matrix where each row is a sample and each column is roll, pitch, or yaw in millimeters; *t* indicates time and *t+1* indicates the subsequent sample.

$$\begin{array}{ccc} & & atan2d(norm(cross(rpy(t,:),rpy(t+1,:))),\\ & & \;dot(rpy(t,:),rpy(t+1,:))) \end{array}$$

As a measure of head stability, the rotational velocity was then calculated by taking the difference in angular rotation between subsequent samples divided by the change in time between samples. For each individual, rotational velocities exceeding the 99^th^ percentile for that subject at that age level were replaced with NaNs in Matlab and excluded from further analysis.

As the rotational velocity data were captured at 60 Hz and the eye-tracking data was captured at 30 Hz, the rotational velocity was downsampled to 30 Hz to accommodate analyses between the sensors. Data were downsampled using cubic smoothing spline interpolation with *csaps* in Matlab. A smoothing parameter of 1 was used, resulting in minimal smoothing.

**A baseline calculation** of the rotational velocity of the head was made for each subject by randomly choosing portions of time when the infant was not looking to the objects in play and was exhibiting gaze that was centered within a 20° radius of the center of the head camera image. The median of this randomly selected baseline was taken, and a 95% bootstrapped confidence interval was calculated.

### Correlation between head movements and eye movements

The vestibulo-ocular reflex (VOR) refers to rapid eye-movements of equal magnitude in the opposite direction counter small head movements that stabilize gaze on a target (Ornitz, Kaplan, & Westlake, 1985; Poletti, Aytekin, & Rucci, 2015; Rosander & Von Hofsten, 2000; Weissman, DiScenna, & Leigh, 1989). Although these compensatory movements are not the focus of the present study, they may be embedded at a finer temporal and spatial resolution than the head stabilizations and larger head movements of central interest. To measure the extent of the VOR within a look, repeated Spearman correlations were used to calculate the moment-to-moment correlation between the rotational yaw of the head and eye movements along the x-axis, horizontal gaze movements for every subject at every age level. Analyses were conducted on the 30 Hz eye-tracking data and motion-tracking data downsampled from 60 Hz to 30 Hz. An algorithm was constructed to calculate the Spearman correlation on the first 500 ms of data (15 data points). The *r* value and *p* value were stored, the bin advanced one data sample, and the correlation was estimated again. This was repeated until the end of the time series was reached.

To determine whether the correlation between the head and eyes exceeded chance, a bootstrapped significance test was conducted. For each of the 1000 permutations, a number of random looks was chosen for each session equal in number and duration to the looks exhibited. Randomly selected looks were binned into 500 ms bins and stored. At the end of the simulation, the 2.5 and 97.5 percentiles of each bin were calculated.

For both of the above calculations, only *r* values that had *p* values less than or equal to 0.01 were included in subsequent analyses.

### Statistical approach

For all the analyses reported in this article, the alpha level was set at 0.01 to minimize the likelihood of false-positive results. The *p* values for each conducted analysis were corrected for multiple comparisons using the Bonferroni-Holm correction (Holm, 1979). Using *lmefit* in Matlab, linear mixed effects (LME) models were constructed for each dependent measure. Dependent measures were as follows: the proportion of time looking to objects, the number of looks to objects, the proportion of looks greater than or equal to three seconds in duration, the median distance of gaze to the center of the head-camera image, the proportion of gaze within a 10° radius of the center of the head camera image, the proportion of gaze within a 20° radius of the center of the head camera image, the proportion of fast head movements, and the proportion of slow head movements. Subject identity and total number of trials, or trial number, were included as a random effect, and infant age level was included as a fixed effect. The formula for these LME were as follows:

$$\begin{array}{ccc} & & dependent\;variable∼age+(1|subject\;identity)\\ & & \;+\;(1|number\;of\;trials) \end{array}$$

Main effects were determined by running an analysis of variance on the LME.

## Results

### Age-related changes in look durations

**Figure 2.**
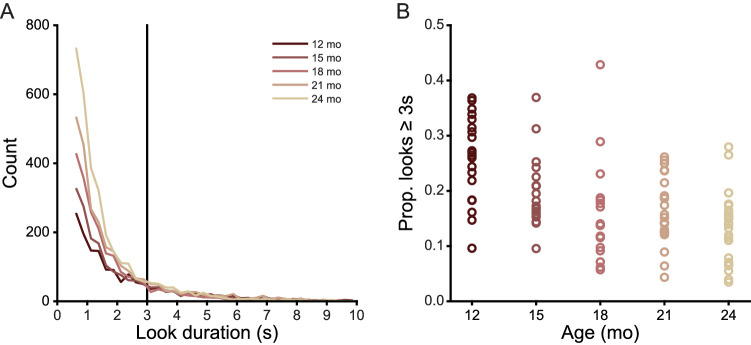
Look duration decreases from 12 to 24 months. (A) Histograms showing the distribution of look durations less than 10 seconds at each age level. Vertical black line indicates the three-second cutoff for short and long looks. (B) Proportion of looks greater than or equal to three seconds in duration for each individual in each age group.

During the six-minute play sessions, children spent a median of 57.12% (*SD* 3.19%, minimum 52.63%, maximum 59.59%) of session time looking to one of the three play objects. An LME revealed no main effect of age on the proportion of time infants looked to objects (*F*(4, 102) = 2.449, *p* = ns). The total number of analyzed frames with gaze directed to an object was 647,698. The total number of looks to an object was 11,055 with the minimum look duration being 15 frames (500 ms). Table 2 provides the median and standard deviation of the proportion of time spent looking at objects and the number of looks to an object for each age level. Although the proportion of time spent looking at the objects did not vary with the age of the infant, the number of looks did (LME, *F*(4, 102) = 4.464, *p* < 0.003), as older infants produced more short looks and younger infants more long looks, a well-known developmental change during this age period (Bronson, 1991; Colombo & Mitchell, 1990; Helo et al., 2016; Wass & Smith, 2014). Figure 2 shows the frequency distribution of look durations less than or equal to 10 seconds in duration, grouped into 500 ms bins. The data included in these graphs include 98.91% of the data analyzed below. Wilcoxon rank sum tests of subsequent age groups revealed look durations become more skewed (proportionally more short looks) with increasing age from 12 to 15 months (*Z* = 5.289, *p* < 0.0001), 15 to 18 months (*Z* = 3.132, *p* < 0.004), and 21 to 24 months of age (*Z* = 5.078, *p* < 0.0001). There was no difference in look duration from 18 to 21 months of age (*Z* = −0.592, *p* = ns). Research on infant visual attention often divides looks into short and long durations (Ruff & Lawson, 1990; Suarez-Rivera, Smith, & Yu, 2019; Wass, Clackson, Georgieva, Brightman, Nutbrown, & Leong, 2018; Wass, Noreika, Georgieva, Clackson, Brightman, Nutbrown, Covarrubias, Leong, 2018; Yu & Smith, 2016; Yu et al., 2019; Yuan, Xu, Yu, & Smith, 2019) using the threshold of a look three seconds or longer for defining long looks. This threshold is near the flexion point in the frequency distribution for all ages (Ruff & Lawson, 1990; Suarez-Rivera et al., 2019; Wass, Clackson, et al., 2018; Wass, Noreika, et al., 2018; Yu & Smith, 2016; Yu et al., 2019; Yuan et al., 2019). As shown in Figure 2B, proportional frequency of long looks, not just the overall durations, also decline with age (LME, *F*(1, 104) = 11.224, *p* < 0.0001). Earlier studies based on human coding of look durations (Ruff & Lawson, 1990) were interpreted as showing steady increases in the frequency of long looks. The more precise measures of the present study suggest that this is not the case.

**Table 2.** Proportion of looking time to objects.

	Proportion looking time to objects	Number of looks to objects
Age level	Median (SD)	Median (SD)
12 months	0.596 (0.097)	80 (29.328)
15 months	0.571 (0.123)	88 (33.038)
18 months	0.526 (0.112)	117 (35.684)
21 months	0.595 (0.129)	116 (32.473)
24 months	0.538 (0.129)	118 (41.745)

### Gaze to the center of the head-centered field of view

**Figure 3.**
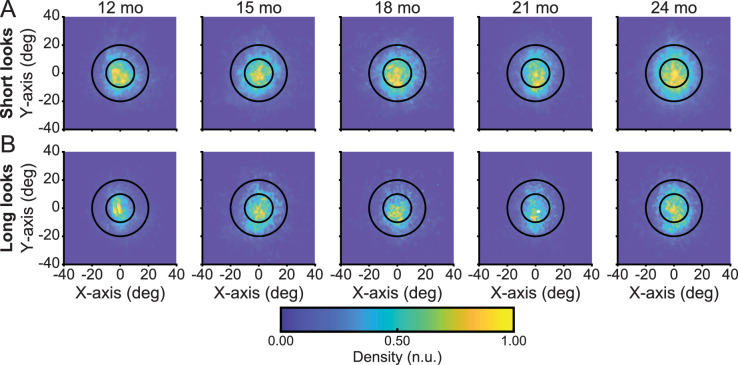
A bias to look at objects with the head and eyes aligned. (A, B) Multivariate kernel density estimates of the accumulated x- and y-coordinates of eye gaze using a head-mounted eye-tracker for gaze to novel objects where (A) looks are shorter than three seconds and (B) looks are equal to or longer than three seconds at 12, 15, 18, 21, and 24 months of age. Inner circles encompass a 10^o^ radius from the center of the head camera image whereas outer circles encompass a 20^o^ radius from the center of the head camera image. Color indicates density of the distribution, with more yellow colors indicating greater density.

Figure 3 shows the distribution of frame-by-frame gaze to objects within the head-centered image for both short and long looks, respectively. As is apparent, both short and long looks are characterized by gaze to the center of the head-centered image. A linear mixed effects model revealed the median distance of gaze points to the center of the head-camera image did not vary as a function of age (*F*(4, 204) = 1.939, *p* = ns) or duration (*F*(1, 204) = 3.593, *p* = ns) and there was no interaction between these factors (*F*(1, 204) = 0.509, *p* = ns). Supplementary Table S1 provides the median and standard deviation of the distance of gaze points to the center of the head-camera image for each age level for both long and short looks. Supplementary Table S2 provides the total number and proportion of data points that fell within a radius of 10° and 20° from the center for each age level. The proportion of gaze points within these two defined regions do not vary as a function of age (LME, 10° radius *F*(4, 204) = 1.805, *p* = ns; LME, 20° radius *F*(4, 204) = 2.974, *p* = ns) nor look duration (LME, 10° radius *F*(1, 204) = 5.318, *p* = ns; 20° radius *F*(1, 204) = 2.803, *p* = ns), and there were no interactions. Across ages, over 34% of gaze fell within 10° of center and more than 78% fell within the 20° radius, indicating the narrow and centered range of gaze to objects within the head camera image. Thus the present findings show what is being consistently observed in studies of ego-centric vision and freely moving perceivers of all ages: a strong bias for looking with head and eyes generally pointed in the same direction.

### Decreased head movement within a look

**Figure 4.**
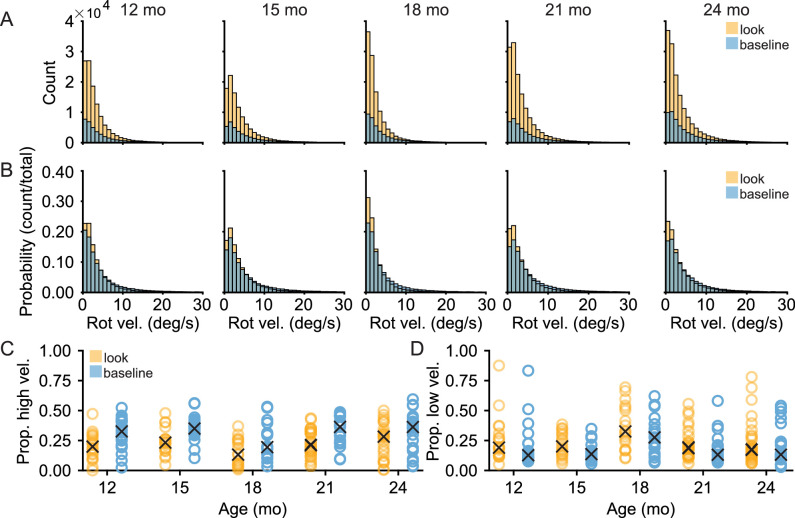
Centered gaze lowers head movements. (A, B) Histograms demonstrating the (A) count and (B) probability distribution of rotational velocity for looks to objects (amber) and looks to targets that were not one of the three play objects or the caregiver's face (blue) at each age level. (C) The proportion of the head's rotational velocity during a look which exceeds the seventy-fifth percentile of the rotational velocity in the observed dataset for every subject at each age level with “X” representing the median proportion for that age level. (D) The proportion of the head's rotational velocity during a look that is slower than the twenty-fifth percentile of the rotational velocity in the observed dataset for every subject at each age level with “X” representing the median proportion for that age level.

Maintained looks to an object within the center of the head-centered field of view imply the coordination of the head and eyes, and thus some limitation on head movements. Figures 4A and 4B show histograms of the head's rotational velocity when infants were looking to objects compared to a baseline where infants exhibited a centered head, with gaze within 20° of the center of the head camera image but were not looking to objects (method of calculating baseline defined in Methods). Histograms include rotational velocity up to 30°/s, which encompasses 99.98% of the observed data. Comparisons of the whole distributions yielded reliable differences between the rotational velocity of the head while looking at objects compared to baseline for each age level (Wilcoxon rank sum test, minimum *Z* = 31.821, maximum *Z* = 54.004, all Bonferroni-Holm corrected *p* < 0.0001). As shown in Figure 4C, the frequency of fast head movements, defined as movements exceeding the 75th percentile of rotational velocity observed in the dataset (5.283°/s) was proportionally greater when infants were exhibiting a centered gaze but not looking at objects than when they were looking at an object (LME, *F*(1, 204) = 9.113, *p* < 0.003) with no main effect of age (*F*(4, 204) = 2.866, *p* = ns) and there was no interaction between looking target and age (*F*(4, 204) = 0.230, *p* = ns). Additionally, as shown in Figure 4D, the frequency of slow head movements, defined as less than the 25th percentile of rotational velocity observed (1.147°/s), was comparable between conditions when infants were looking at objects than when they were not (LME, *F*(1, 204) = 1.373, *p* = ns) with no main effect of age (*F*(2, 204) = 2.572, *p* = ns) nor an interaction between looking target and age (*F*(2, 204) = 0.071, *p* = ns). Relative to comparably centered looks, looks to objects exhibited fewer fast head movements and a comparable amount of slower head movements across all ages.

**Figure 5.**
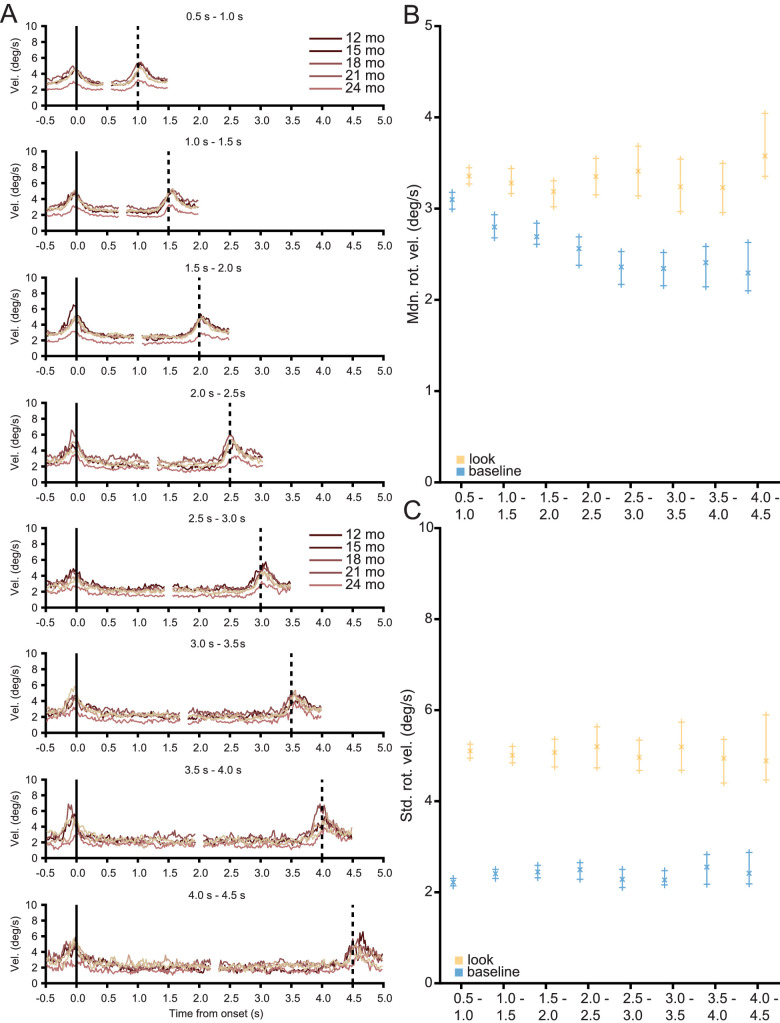
Head stability is a function of look duration. (A) Median rotational velocity traces aligned to the onset (vertical solid black line) and offset (vertical dotted black line) of a look with lighter colors indicating older groups. Traces are aligned to the onset and offset of a look, beginning 500 ms before the onset and ending 500 ms after the offset of the look. Because instances of looks to an object vary in duration, the rotational velocity traces were binned into 500 ms bins. (B, C) The (B) median and (C) standard deviation of the rotational velocity of the head for the binned looks (amber) with a calculated baseline (blue). Error bars indicate 95% bootstrapped confidence intervals.

For all look durations, at all ages, head movements markedly decrease after the onset of a look. As the duration of each look is variable, look duration was binned into 500 ms bins, up to a maximum of 4.5 seconds. Such a cutoff includes 99.95% of the observed data. Figure 5A shows the median rotational velocity of the head aligned to the onset and offset of a look. Velocity profiles begin 500 ms before the onset of a look and end 500 ms after the offset of a look. Supplementary Table S3 lists the number and proportion of looks in each bin for each age level. Across all look durations, looks begin with a brief change in velocity followed by a slowing of the head before the look ends with another brief change in velocity at the look's offset. Figure 5B shows the median rotational velocity of the head for the looks in each of the bins in Figure 5A compared to the baseline rotational velocity of the head (method of calculating baseline defined in Methods). Figure 5C shows the standard deviation of rotational velocity of the head for looks to objects and for the baseline. Baseline encompasses moments when infants were not looking to either of the three objects and their gaze was within 20° of the center of the head camera image. Error bars for both the baseline and observed rotational velocity indicate the 95% bootstrapped confidence interval. The median rotational velocity of the head was lower during a look than baseline for every bin (Wilcoxon rank sum test, minimum *Z*: −8.102 maximum *Z*: −4.325, all Bonferroni-Holm corrected *p* < 0.0001). The standard deviation of the rotational velocity of the head was lower during a look than baseline for every bin (Wilcoxon rank sum test, minimum *Z*: −33.643 maximum *Z*: −7.940, all Bonferroni-Holm corrected *p* < 0.0001). In sum, infants between the ages of 12 and 24 months consistently and uniformly look to objects with their eyes and head aligned and they maintain alignment throughout the look by slowing their head movement and minimizing its variability.

### The function of a stabilized head

**Figure 6.**
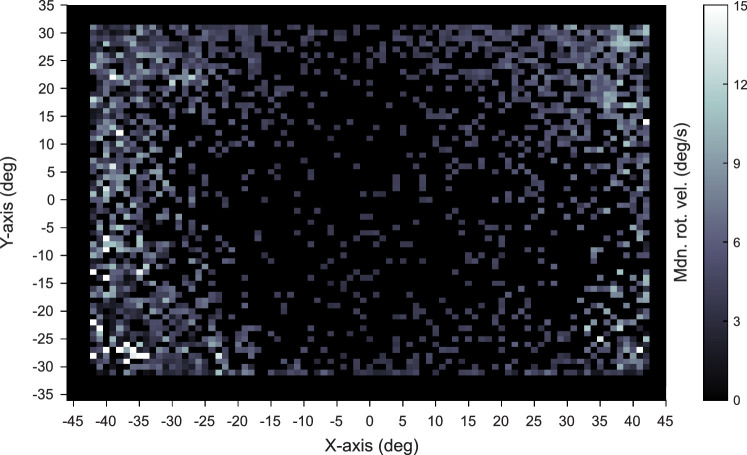
Head velocity is slower when gaze is at the center of the head camera image. Median head movement for each position of the eye in the head camera image calculated across all subjects at all ages. Brighter colors indicate a greater head velocity.

During a look, infants make rapid head movements and minimize variability in head movements. This stabilization does not imply a complete stillness of the head. At no point in time did any subject at any age exhibit head movement that was 0°/s. As demonstrated in the distributions of rotational velocity in Figures 4A and B, head movement is continuous, and there is no sharp divide between a still and not-still head. The decreased movement characteristic of looks to an object, however, are associated with the spatial location of gaze in the field of view. The head camera image was divided into bins 1 visual degree in height and width and the median rotational velocity of the head was calculated for each bin. Figure 6 shows the median rotational velocity of the head for each eye position in the head camera image across all ages. Gaze to the center of the head camera image coincides with a low head velocity while gaze to the periphery of the head camera image coincides with high velocity head movements. Thus, a slower-moving more stabilized head is strongly associated with the centering of gaze within a head-centered field of view.

### A measurable vestibulo-ocular reflex?

Does VOR provide a measurable contribution to the stabilization of the head and eyes during a look? If VOR is present and playing a role within looks to an object, there should be a negative correlation between the horizontal direction of head and eye movements. In an attempt to measure the possible contribution of this reflex in active naturalistic viewing, the yaw rotation of head movement was correlated to horizontal movement of the eyes during looks. Moments when the eyes and head moved in the same direction resulted in positive *r* values while moments when the eyes and head moved in the opposite direction resulted in negative *r* values. For every individual session, the moment-to-moment Spearman correlation between the head and eyes was calculated in successive, overlapping 500 ms bins during a maintained look to an object. As the analysis was conducted on the 30 Hz eye-tracking data and the downsampled motion capture data, 500 ms corresponds to 15 data points. The moment-to-moment *r* value was then calculated for every look, up to 4.5 seconds in duration, at every age and binned into 500 ms bin durations. For the duration of the look, while the correlation between the head and eyes changes over time, the extent of the correlation did not exceed chance. This lack of a measurable VOR in natural viewing is consistent with previous reports (Agtzidis, Startsev, & Dorr, 2019; Fuller, 1996; Meyer, O'Keefe, & Poort, 2020; Tatler, 2007; Tseng, Carmi, Cameron, Munoz, & Itti, 2009; Wang, Koch, Holmqvist, & Alexa, 2018) that were also unable to detect VOR in natural vision. Thus the role of VOR in active natural viewing remains an unanswered question in need of further study and better measurement approaches.

## Discussion

During play, toddlers look to objects with a stilled head but rapidly move their head to begin and end a look. Looking at an object with the head and eyes aligned appears to be the default mode for both short and long looks and does not vary with age during the period between the first and second birthday. For toddlers, gaze sustained on an object for any duration begins with the rapid movement of the head and eye to the object which is then maintained by limited head movement with the centering of gaze within a head-centered frame of reference. The look ends with another rapid movement of the head and eyes. These findings contribute to the understanding of visual attention in freely moving perceivers in the context of their own self-generated purposeful behavior, which is the context of everyday vision. Within this context, a suite of behaviors appears to form a complex interdependent system of shifting both gaze and head in the same direction then maintaining gaze on an object with limited head movements such that the looked-to object is centered in a head-centered field of view.

The head and eyes can and do move independently: What, then, is the function of the observed strong coordination of the head and eye movement at the start of a look, the joint stabilization of eye and head direction to the attended object such that gaze is centered within the head-centered view, and the synchronous shift of both head and eyes to end a look? Both behavioral (Cicchini, Valsecchi, & De'Sperati, 2008; Corneil & Munoz, 2014; Khan, Blohm, McPeek, & Lefèvre, 2009) and neural (Gandhi & Katnani, 2011; Ignashchenkova, Dicke, Haarmeier, & Thier, 2004; Müller, Philiastides, & Newsome, 2005; Stryker & Schiller, 1975; Walton, Bechara, & Gandhi, 2007) evidence indicates that the networks that plan motor behaviors (Desimone & Duncan, 1995; Miller & Cohen, 2001; Miyake & Friedman, 2012) overlap with the networks that internally control the spatial direction of visual attention (Cicchini et al., 2008; Corneil & Munoz, 2014; Khan et al., 2009). Planning and executing the independent movement of different body parts—the head, eyes, and hands—requires the coordination of multiple spatial reference frames (Galati, Pelle, Berthoz, & Committeri, 2010; Lappi, 2016; Schlicht & Schrater, 2007). For example, in looking and reaching to an object, the actor must coordinate the reference frame for the eye by moving gaze from the current eye position to the target and for the hand by moving the hand from its current position, which is different from the eye, to the target. In freely-moving individuals, the reference frames for the eyes, head, torso, and hand must continuously be coordinated (Badde, Röder, & Heed, 2015; Bosco, Piserchia, & Fattori, 2017; Crollen et al., 2017; Crollen, Spruyt, Mahau, Bottini, & Collignon, 2019; Pouget, Deneve, & Duhamel, 2002; Tagliabue & McIntyre, 2014). Considerable research shows this coordination is difficult and imposes a measurable computational burden not just on action but also on visual attention with effects on the detection, discrimination and location of visual events. For example, in adults, the misalignment of the head and eyes destabilizes and disrupts gaze relative to the aligned head and eyes (Einhäuser, Schumann, Bardins, Bartl, Böning, Schneider, & König, 2007; Flanders, Daghestani, & Berthoz, 1999; Thaler & Todd, 2009) and goal-directed bodily actions become less spatially precise when the head and eyes point in different directions.

Between their first and second birthday, toddlers are in the midst of mastering many new bodily movements and skills. Considerable research shows that toddlers decrease the degrees of freedom in frames of reference for body movements by limiting or aligning the movement of different body parts when initially walking, carrying objects, or bending over to pick up an object (Claxton et al., 2013; Claxton et al., 2012; Claxton et al., 2014; Smith & Thelen, 1996). Looking is a motor behavior. Just as toddlers planning and controlling of other actions benefits from synergistic movements, so may the spatially coordinated head and eyes support visual attention. Gaze to the midline of the head and body is the positional resting state, and it may take more energy to maintain gaze in eccentric orbital positions, the eyes will naturally return to the center. But an aligned and stabilized head and eyes for the duration of a look to a target may also not just be easy but highly functional by limiting coordination and competition among spatial frames of reference (Einhäuser et al., 2007; Flanders et al., 1999; Thaler & Todd, 2009).

The brief changes in rotational velocity of the head at the onset and offset of a look have been described previously, albeit in very young infants, but also may have a key role in toddler visual attention. Infants at three months of age exhibit rapid bursts of body movement preceding gaze shifts during screen-based viewing and these have been shown to facilitate ending a look to one target to shift to another (Robertson, Johnson, Masnick, & Weiss, 2007) because young infants have considerable difficulty in disengaging from an attended target. Young infants who exhibit less coordinated bursts in movement during screen-based viewings went on to develop deficits in attention (Friedman, Watamura, & Robertson, 2005). Indeed, similar disruptions in sensory-motor coordination are exhibited by premature infants (Berger, Harbourne, & Guallpa Lliguichuzhca, 2019) and infants with several developmental disorders (Hartman, Houwen, Scherder, & Visscher, 2010; Proudlock & Gottlob, 2007). Toddlers with more well-developed control of eye, head, and body movements may well use head movements to purposely break gaze, a hypothesis worthy of future study.

Toddlers’ ability to maintain a look to an individual object during active object play strongly predicts later developments in executive function and self-regulation and has been proposed to be causally related to those developments (Brandes-Aitken et al., 2019; Fisher, 2019; Rosen et al., 2019; Werchan & Amso, 2017; Yu & Smith, 2016). The origins of individual differences in sustained attention has not been identified (see Rosen et al., 2019). The goal here was to determine the mechanics of the behaviors—both the head and eyes—that underlie continuous looks to an object as a first step to understanding potential sources of individual differences. Uncontrolled body movements and specifically head movements have been linked to poor attentional control in older children (Friedman et al., 2005; Hartman et al., 2010; Proudlock & Gottlob, 2007) suggesting the integrative hypothesis that disruptions in sensory-motor coordination of eyes and head lead to disrupted attentional abilities. For example, toddlers diagnosed with autism spectrum disorders sometimes exhibit difficulties in maintaining the midline position of the head during active attentional tasks (Dawson, Campbell, Hashemi, Lippmann, Smith, Carpenter, Egger, Espinosa, Vermeer, Baker, Sapiro, 2018; Martin, Hammal, Ren, Cohn, Cassell, Ogihara, Britton, Gutierrez, Messinger, 2018), a bias strongly evident in typically developing toddlers (Bambach et al., 2018; Bambach et al., 2016). Difficulties in early head and trunk control are also exhibited by children with Down syndrome (Cardoso, De Campos, Dos Santos, Santos, & Rocha, 2015; Rast & Harris, 2008), language delays (Vuijk, Hartman, Scherder, & Visscher, 2010), and other cognitive disorders (Visscher, Houwen, Scherder, Moolenaar, & Hartman, 2007). Many of these disorders occur with concomitant deficits in the control of visual attention.

In conclusion, the present study provides evidence on eye and head coordination in infant looking behavior during active self-generated interactions with objects, the context of children's everyday vision and visual learning. There is much that is not known about looking behavior in this context. The present results provide a first step by showing a tight coordination of head and eyes during toddlers’ sustained looks to objects.

## Supplementary Material

Supplement 1
