# Supplementary material for: Head and eyes: Looking behavior in 12- to 24-month-old infants
Source: J Vis. 2021 Aug 17;21(8):18. doi: 10.1167/jov.21.8.18 (PMC8375006; doi:10.1167/jov.21.8.18)
Supplement: Supplement 1 [file jovi-21-8-18_s001.pdf]

Age	Long Look	Short Look
12 Months	10.957 (2.518)	11.790 (2.426)
15 Months	12.314 (2.821)	12.376 (2.783)
18 Months	11.501 (2.472)	12.549 (1.938)
21 Months	10.895 (2.111)	11.739 (1.653)
24 Months	11.628 (2.105)	13.333 (2.025)
**Overall**	**11.550 (2.409)**	**12.471 (2.212)**

Supplementary Table 1. *Distance to the center of the head camera image for the data plotted in Figure 3.* For each age level, the median distance to the center of the head camera image for each age level and for both short and long looks is provided with standard deviations in parentheses.

Age	Count					Proportion		
	0-10° Radius	10-20° Radius	Inside 20°	Outside 20°	Total	0-10° Radius	10-20° Radius	Inside 20°
12 Months	52,082	52,408	104,490	23,360	127,850	0.407	0.410	0.817
15 Months	38,250	51,842	90,092	28,188	118,280	0.323	0.438	0.762
18 Months	44,414	57,067	101,481	33,057	134,538	0.330	0.424	0.754
21 Months	65,027	79,074	144,101	30,058	174,159	0.373	0.454	0.827
24 Months	55,810	81,190	137,000	43,847	180,847	0.309	0.449	0.758
**Overall**	**255,583**	**321,581**	**577,164**	**158,510**	**735,674**	**0.347**	**0.437**	**0.785**

Supplementary Table 2. *Breakdown of data plotted in the kernel density estimates in Figure 3.* For each age level, the amount of data within each of the circles plotted in Figures 2a and 2b as well as the proportion of data that falls in each circle.

Age	0.5 – 1. sec	1.0 – 1.5 sec	1.5 – 2.0 sec	2.0 – 2.5 sec	2.5 – 3.0 sec	3.0 – 3.5 sec	3.5 – 4.0 sec	4.0 – 4.5 sec
12 Months	441	286	185	132	105	81	58	43
15 Months	591	358	194	137	109	84	58	40
18 Months	782	474	268	156	122	65	57	37
21 Months	983	497	299	196	136	98	62	46
24 Months	1,316	713	338	227	118	106	80	58
**Overall**	**4,113**	**2,328**	**1,284**	**848**	**590**	**434**	**315**	**224**
12 Months	0.331	0.215	0.139	0.099	0.079	0.061	0.044	0.032
15 Months	0.376	0.228	0.123	0.087	0.069	0.053	0.037	0.025
18 Months	0.399	0.242	0.137	0.080	0.062	0.033	0.029	0.019
21 Months	0.424	0.215	0.129	0.085	0.059	0.042	0.027	0.020
24 Months	0.445	0.241	0.114	0.077	0.004	0.036	0.027	0.020

Supplementary Table 3. *Breakdown of data plotted in Figure 5.* For each age level, the number of looks within each bin for each age level across all participants (at top) and the proportion of looks within each bin normalized for each age group.
